# Effects of occlusion pressure on hemodynamic responses recorded by near-infrared spectroscopy across two visits

**DOI:** 10.3389/fphys.2024.1441239

**Published:** 2024-09-11

**Authors:** Julien Desanlis, Dan Gordon, Chloe French, Camille Calveyrac, François Cottin, Marie Gernigon

**Affiliations:** ^1^ CIAMS, Université Paris-Saclay, Orsay, France; ^2^ CIAMS, Université d’Orléans, Orléans, France; ^3^ Cambridge Centre for Sport and Exercise Sciences, Anglia Ruskin University, Cambridge, United Kingdom

**Keywords:** remote ischemic preconditioning, arterial occlusion, muscle oxygenation, blood flow restriction, muscle ischemia, NIRS

## Abstract

Ischemic Preconditioning (IPC) has emerged as a promising approach to mitigate the impact of hypoxia on physiological functions. However, the heterogeneity of occlusion pressures for inducing arterial occlusion has led to inconsistent hemodynamic outcomes across studies. This study aims to evaluate the peripheral hemodynamic responses to partial and total blood-flow occlusions on the left arm at rest, using absolute or individualized pressures, on two occasions. Thirty-five young males volunteered to participate in this study. IPC procedure (3 × 7-min) was performed on the left upper arm with cuff pressures at 50 mmHg (G1), 50 mmHg over the systolic blood pressure (SBP + 50 mmHg) (G2) or 250 mmHg (G3). NIRS-derived parameters were assessed for each occlusion and reperfusion phase in the brachioradialis. Results showed a significantly lower magnitude of deoxygenation (TSIAUC) for G1 compared to G2 (−1959.2 ± 1417.4 vs. −10908.1 ± 1607.5, *P* < 0.001) and G3 -1959.2 ± 1417.4 vs. −11079.3 ± 1828.1, *P* < 0.001), without differences between G2 and G3. However, G3 showed a significantly faster reoxygenation only for tissue saturation index (TSI_slope_) compared to G2 (1.3 ± 0.1 vs. 1.0 ± 0.2, *P* = 0.010), but without differences in the speed of recovery of deoxyhemoglobin [(HHb) slope], or in the magnitude of post-occlusive hyperemia (PORH). Besides TSI reoxygenation speed, G2 and G3 elicit comparable resting hemodynamic responses measured by NIRS. Thus, this study highlights the practicality and effectiveness of using relative occlusion pressures based on systolic blood pressure (SBP) rather than relying on excessively high absolute pressures.

## 1 Introduction

Ischemic Preconditioning (IPC) is a clinical procedure that protects organs against ischemia-reperfusion injury by inducing transient cycles of arterial blood flow occlusion and reperfusion (Kloner, 2009; O’Brien and Jacobs, 2021). Originally used to prevent irreversible injuries of coronary artery occlusion (Murry et al., 1986); IPC is one of the most powerful and reproducible phenomena in cardioprotection (Kloner, 2009). However, the original invasive procedure consists of inflating and deflating a balloon placed in the artery or clamping and unclamping the artery, with the possibility of further complications, such as atherosclerotic emboli in the coronaries (Kloner, 2009). To address these risks, the effects of repeated remote, non-invasive brief ischemia’s have been investigated previously to overcome the invasive procedural shortcomings. In this approach, a pressure cuff is applied to the upper or lower limb and inflated above systolic blood pressure (SBP) to occlude the remote vascular bed (Przyklenk et al., 1993). This method avoids the complications of invasive IPC procedures while being equally effective (Kloner, 2009; Przyklenk et al., 1993). Safe and cost-effective, IPC has been transposed to sport sciences to prevent the deleterious effects of hypoxia on performance (Marocolo et al., 2016). Effects of IPC on neuromuscular, endothelial and oxidative functions, including enhancing angiogenesis (Gu et al., 2021; Michiels, 2003; Rajendran et al., 2013) and cutaneous microcirculation (Gerovasili et al., 2009; Gomez et al., 2008; Kolbenschlag et al., 2017; Kraemer et al., 2011; Rosenberry and Nelson, 2020; Sogorski et al., 2021), have been widely documented (Paradis-Deschênes, 2021). Although IPC’s effectiveness in clinical guidelines has been well established, the significant effects of IPC on performance are still debated (Marocolo et al., 2016), particularly regarding assessment methods (Marocolo et al., 2019; Paradis-Deschênes, 2021).

Mechanisms of IPC currently need to be better understood. The literature suggests that the beneficial effects of IPC operate through neuronal pathways, humoral substances released into the systemic circulation or peripheral hemodynamic responses (Kloner, 2009; O’Brien and Jacobs, 2021; Paradis-Deschênes et al., 2016). Between 3 and 5 cycles of 5–10 min are recommended to induce positive effects of IPC on performance and avoid deleterious adverse effects (Johnsen et al., 2016; Kolbenschlag et al., 2017; Sogorski et al., 2021), such as skin or muscle injuries, or “hyper-conditioning” (Kam et al., 2001; Whittaker and Przyklenk, 2014). Thus, inflating cuffs to the required pressure is essential to preserve participant integrity. In the literature, two different methods of determining cuff pressure are used: absolute or individualized based on systolic blood pressure (SBP) (O’Brien and Jacobs, 2021). For absolute pressures, the same pressure is applied for each participant implying that the selected pressure is high enough to occlude the blood flow for every participant. Absolute pressures often range between 180 and 250 mmHg (O’Brien and Jacobs, 2021), for the upper limb, which represents between 130% and 210% of the SBP for a typical 120 mmHg SBP (Hardy et al., 2021). Alternatively, upper limb arterial occlusion pressure could be based on SBP, when using the same cuffs for BP estimation and IPC intervention (Loenneke et al., 2015). Individualized pressures used for upper limb arterial occlusion range between 15 and 50 mmHg over the SBP, to induce arterial occlusion (O’Brien and Jacobs, 2021). However, inflating the cuff only up to 15 mmHg over the SBP (SBP + 15 mmHg) value may be insufficient to achieve the occlusion of certain deep arterioles (Reid et al., 1983; Sharma et al., 2014). Due to factors such as limb positioning and non-uniform cuff inflation, it is necessary to inflate the cuff to a minimum pressure of 50 mmHg over the SBP (SBP + 50 mmHg) to reach complete blood flow occlusion (Reid et al., 1983; Sharma et al., 2014). The use of individualized pressures for IPC appears to be safer for the participant by reducing the pressure used in a normotensive population (Reid et al., 1983), but may also allow complete occlusion for participants with high SBP. The same approach of choosing individualized pressures based on SBP is also recommended in Blood Flow Restriction (BFR) training (Loenneke et al., 2015; McEwen et al., 2019). The recommended pressure for BFR training ranges from 40% to 80% of AOP (Patterson et al., 2019), with 40% of AOP showing similar effects on muscle deoxygenation and activation as 80% of AOP (Gray et al., 2023). Thus, selecting an absolute or an individualized pressure to occlude the blood flow, may induce variations in the magnitude of hemodynamic disturbances. Both at rest and during exercise, near-infrared spectroscopy (NIRS) could be used to assess peripheral hemodynamic responses (Van Beekvelt, 2002). NIRS provides non-invasive estimations of haemoglobin concentration changes, giving information on muscular tissue oxygenation (Ferrari et al., 2011; Perrey and Ferrari, 2018). The reperfusion rate after ischemia allows differentiation between trained and untrained (Perrey and Ferrari, 2018) as well as healthy and diseased populations (Hamaoka et al., 2007). Additionally, sex-related differences emerge when desaturation levels are matched, with men exhibiting greater PORH than women, potentially due to phenotypic characteristics (Keller et al., 2023). These sex-based differences in muscle oxygenation responses, as measured by NIRS, could highlight variations across different groups (Keller et al., 2023). During exercise, muscular deoxygenation and oxygen extraction after the IPC procedure have been investigated (Patterson et al., 2015; Tanaka et al., 2016). NIRS technology was found to be reliable for intra-session measurements, but cautiousness is needed when comparing measurements between sessions at rest (Desanlis et al., 2022).

Heterogeneous results regarding the ergogenic aid of IPC have been reported in the literature, attributed to a lack of standardization in IPC protocols, especially concerning occlusion pressure (O’Brien and Jacobs, 2021). IPC has been found effective in enhancing athletic performance, $\dot{V}O_{2}\max$ , and increasing blood flow and muscle oxygenation during exercise in some studies, but did not yield significant changes in other studies (Marocolo et al., 2016; Marocolo et al., 2019). It may then be of interest to further explain any peripheral dynamic responses assessed by NIRS. When the cuff is inflated and occlude the blood flow totally (arterial occlusion), the O_2_ is consumed leading to decrease in O_2_Hb and TSI and a concomitant increase in HHb (Van Beekvelt, 2002). After a few minutes of occlusion (≈3–5 min), the NIRS signals plateaus, showing the maximal deoxygenation reached (Barstow, 2019; Ferrari et al., 1992; Grassi and Quaresima, 2016). This minimum of TSI is not to confound with a true zero (TSI = 0%), that would require much time and/or the addition of exercise (Barstow, 2019). Upon deflation, the muscle tissue is rapidly reperfused allowing for calculation of reoxygenation slope (Rosenberry and Nelson, 2020; Van Beekvelt, 2002). The reoxygenation reaches a maximum of TSI that exceeds the baseline value and could be quantitatively calculated using the aera under the curve (AUC) method (Agbangla et al., 2017; Barstow, 2019). However, muscle microcirculation is affected not only by arterial occlusion but also by partial blood flow occlusion at rest, which significantly reduces muscle oxygenation (Cherouveim et al., 2021). Thus, this study aimed to evaluate the peripheral hemodynamic responses assessed by NIRS to partial or complete, absolute or individualized blood-flow occlusions on the left arm at rest. We hypothesized that complete arterial occlusion would induce greater deoxygenation and reoxygenation during and after the occlusion but without significant differences between absolute or individualized arterial occlusion pressure since both pressures are high enough to induce complete arterial occlusion.

## 2 Methods

### 2.1 Participants

This study was supported by local institutional ethical approval (CER-Paris-Saclay-2020-006), and written informed consent was provided by all participants. Forty-six young males volunteered to participate. Only males were asked to participate in order to limit experimental bias linked to a sex-related effect in microvascular responses (Chambliss and Shaul, 2002; Kao and Sun, 2015). Fluctuations in circulating oestrogen levels throughout the menstrual cycle impact both vasodilation and blood pressure (Dubey et al., 2002; Mendelsohn and Karas, 2005; Reckelhoff, 2001). Due to the necessity of ensuring consistency between sessions and the inability to evaluate oestrogen levels in our participants, females were excluded from this study.

**FIGURE 1:**
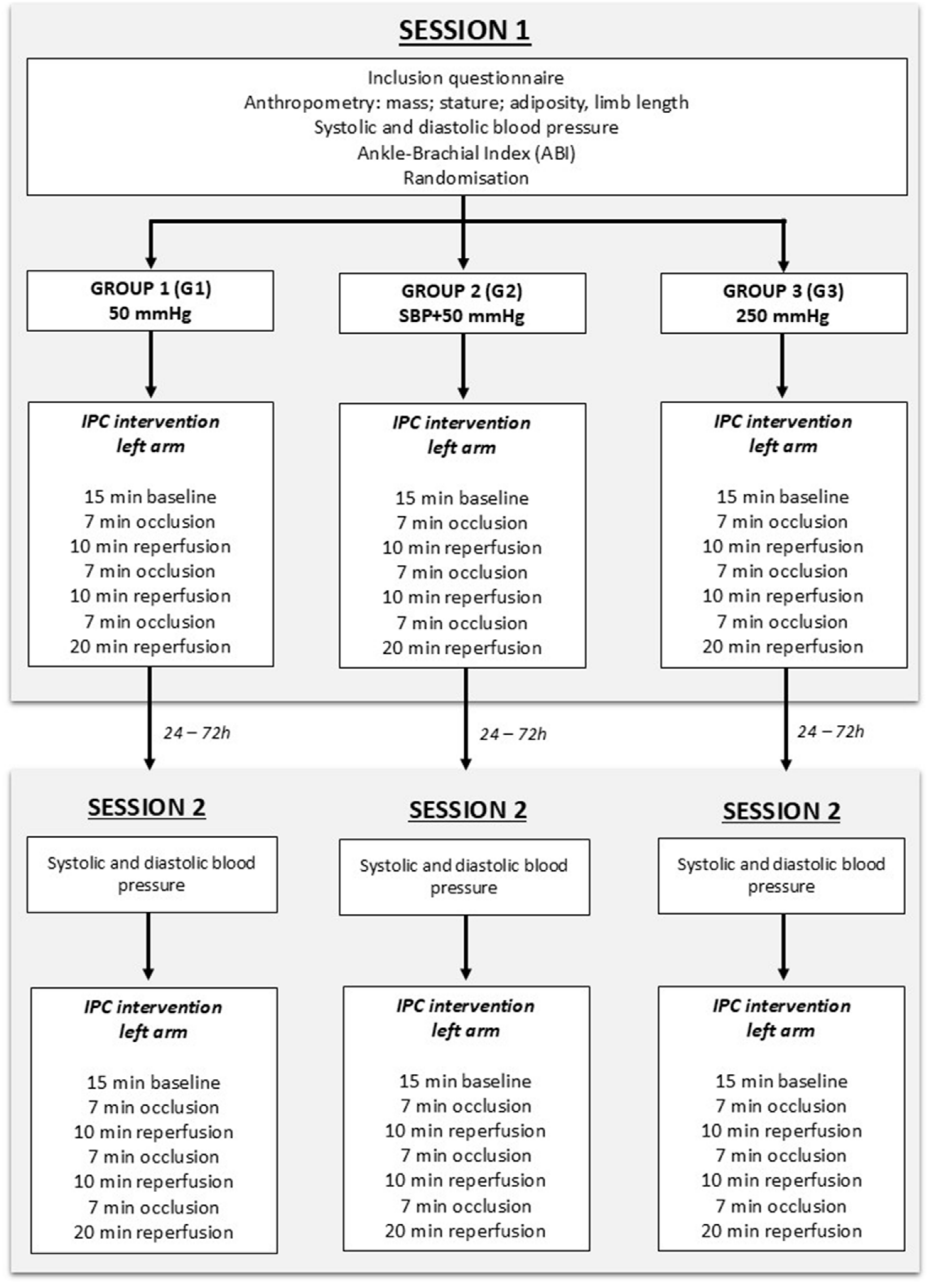
Flowchart of the study design. During the protocol, the participant was supine on a medical couch, with the arm at the level of the heart. The baseline consisted of 15 min of rest, without any occlusion, to establish resting values.

Participants who reported diabetes, hypertension, previous cardiovascular events, and any unordinary vigorous physical activity 24 h before the session (e.g., competition), were excluded from the study. Participants were asked to avoid alcohol intake 24 h before the sessions and caffeine or food intake 2 h before any session. Participants made two separate visits to the laboratory (Figure 1), within 24–72 h. All participants were scheduled for their visits between 9:00 a.m. and 18:30 p.m., ensuring consistency in the timing of both visits for each participant.

Participants were asked by the investigator to provide the type and duration of weekly physical activity, including walking as defined by the World Health Organization (WHO, 2024). Physical activity type (aerobic, resistance, mixed) is reported in Table 1 as a frequency table based on the classification of Howley (2001). The term “mixed” refers to participants who engage in both aerobic and resistance activities. No physical fitness examination was conducted.

**TABLE 1:** Description of environmental and participants’ characteristics.

Group	G1 (50 mmHg)	G2 (SBP + 50 mmHg)	G3 (250 mmHg)	Total
n	9	14	12	35
Age (years)	22 ± 4	22 ± 3	22 ± 4	22 ± 3
Stature (cm)	177 ± 6	176 ± 7	183 ± 8	178 ± 7
Mass (kg)	77 ± 15	73 ± 13	76 ± 7	75 ± 12
Body fat (%)	14.5 ± 5.4	16.6 ± 5.5	12.5 ± 2.8	15 ± 5
Physical activity (h/wk)	5 ± 2	9 ± 7	10 ± 8	8 ± 6.4
Aerobic (%)	75.0	57.1	41.7	55.9
Mixed (%)	12.5	28.6	41.7	29.4
Resistance (%)	12.5	14.3	16.7	14.7
SBP (mmHg)	115 ± 7	116 ± 10	122 ± 9	118 ± 9
DBP (mmHg)	66 ± 7	71 ± 6	68 ± 7	69 ± 7
ABI	1.04 ± 0.08	1.03 ± 0.07	1.03 ± 0.06	1.03 ± 0.07
Temperature (°C)	21.0 ± 1.3	21.8 ± 1.4	20.8 ± 1.7	21.2 ± 1.5
Humidity (%)	38 ± 14	45 ± 13	42 ± 15	43 ± 14
Occlusion pressure	50	166 ± 10	250	

Values are reported as mean ± SD; SBP, systolic blood pressure; DBP, diastolic blood pressure; ABI, ankle to brachial index; G1, group 1; G2, group 2; G3, group 3. Physical activity type (aerobic, resistance, mixed) is reported as a frequency table based on the classification of Howley (2001). The term mixed is used when the participant practices both aerobic- and resistance-based activities.

### 2.2 Study design

During the first session (“S1”), anthropometric measurements (stature, body mass, adiposity, and limb length) and blood pressure were assessed. Then, participants were randomly assigned to one of three groups with different occlusion pressures applied to the left arm: 50 mmHg (G1) (to occlude venous return (Wilkinson and Webb, 2001)), 50 mmHg over the systolic blood pressure (SBP + 50 mmHg, (G2)) and 250 mmHg (G3) (O’Brien and Jacobs, 2021; Sogorski et al., 2021). The protocol consisted of a 15-min baseline period, followed by 3 min × 7 min occlusion phases interspersed with three reperfusion phases of 10, 10 and 20 min where the cuff was deflated (Desanlis et al., 2022), as depicted in Figure 1. Occlusions were induced using a manual sphygmomanometer (Easy 3, Holtex +, Aix-en-Provence, France) and manually adjusted to maintain the selected pressure during the allotted 7-min. Inflation and deflation durations are approximatively 5–8 s. During the whole protocol, the participant was supine on a medical couch, with the arm in a horizontal position at the same level than the heart, to prevent limb position effects on blood flow (Jasperse et al., 2015; Krishnan et al., 2011).

During the second session (S2), participants received the same intervention as in S1, at the same time of day and with the same occlusion pressure, to ensure consistency between sessions. Humidity and temperature were assessed at the beginning of each session by a digital and wireless weather station (Mtraining, France).

### 2.3 Measurements

#### 2.3.1 Skinfolds

Triplicate skinfold thickness measurements were attained from four sites (nondominant subscapular, bicipital, tricipital, and suprailiac) by a single researcher responsible for all skinfolds assessment using Harpenden Skinfold Caliper (Baty International, Wantage, United Kingdom), according to ISAK standards. Skinfold thickness was calculated as the sum of the average values of the four sites. Fat percentage was determined with the correlation table provided by the manufacturer.

#### 2.3.2 Ankle-brachial index (ABI)

ABI was assessed in the supine position by a trained investigator in all participants to verify their peripheral cardiovascular health status (Clark et al., 2011; Sacks et al., 2003). ABI was calculated according to the recommendations of Aboyans et al. (2012) (Equation 1). SBP was assessed with a manual sphygmomanometer (Easy 3, Holtex +, Aix-en-Provence, France) and a manual stethoscope (Classic III, 3M Littman Stethoscopes, Maplewood, United States) for the first measurement of the right brachial artery and with a mini-doppler (Sonotrax Lite, Edan Instruments Inc., Shenzhen, China) for subsequent measures.

$$ABI=\frac{Highest\;ankle\;SBP\;\left(mmHg\right)}{Highest\;brachial\;SBP\;\left(mmHg\right)}$$ (1)

#### 2.3.3 Near-infrared spectroscopy (NIRS)

A wireless NIRS device (PortaMon, Artinis, Elst, Netherlands), connected with Bluetooth with a sampling rate of 10 Hz, was located on the left arm. This is dual-wavelength device (760 and 850 nm), with three pairs of LEDs spaced 30, 35, and 40 mm from the receiver, continuous-wave NIRS system using the modified Lambert-Beer law. It calculates the concentration changes of tissue oxy-, deoxy- and total haemoglobin (O_2_Hb, HHb, tHb, respectively). The tissue saturation index (TSI), expressed in percentage (%) and reflecting the dynamic balance between dioxygen (O_2_) supply and consumption, is calculated as stated in Equation 2:

$$TSI=\left[O_{2}Hb\right]/\left(\left[O_{2}Hb\right]+\left[HHb\right]\right)\times 100$$ (2)

**FIGURE 2:**
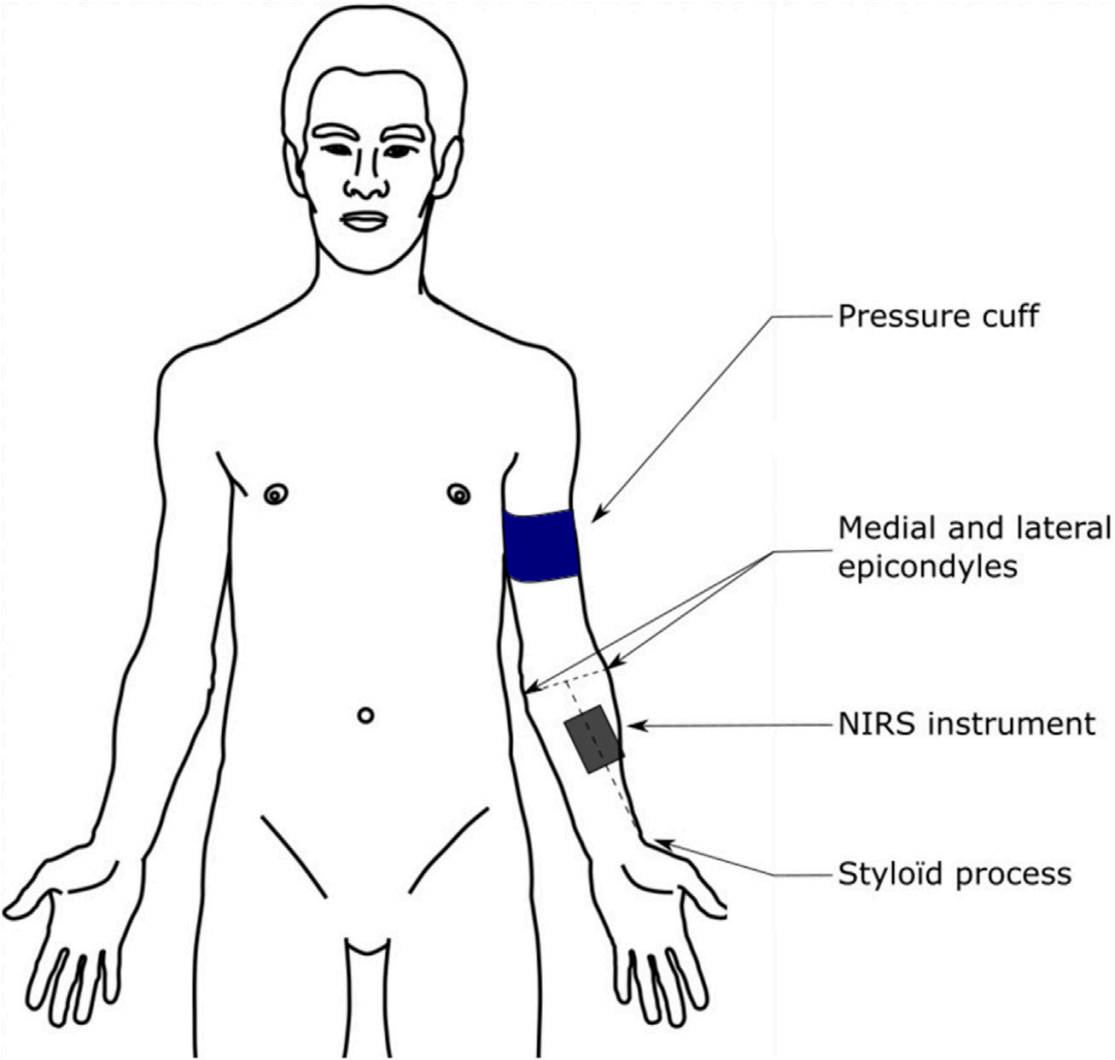
Illustration of the NIRS device placement. Reproduced with permission from Desanlis et al. (2022).

The NIRS device was placed on the brachioradialis muscle, at two-thirds on the line from the styloid process to the central point between the lateral and medial epicondyles (Figure 2), to have consistent device placement for all participants (Barbero et al., 2012). The device was adhered to the limb with double-sided auto-adhesive tape (Coheban, 3M, Cergy-Pontoise, France) and wrapped in black cloth and an elastic bandage to prevent any disturbance due to light interference or unintentional movement.

### 2.4 Data analysis

NIRS data was smoothed using a 10th-order low-pass zero-phase Butterworth filter (cut-off frequency 0.8 Hz) using Pandas software library functions for Python (Python 3.8.8, Python Software Foundation, https://www.python.org) (McKinney, 2018). All data were normalized on the average of the ten central min of the baseline (i.e., t = 150–t = 750 s) and expressed as the magnitude difference with respect to baseline values. Hemoglobin difference (ΔHb), indicating oxygenated blood (Cunniffe et al., 2017), was calculated as the difference between the oxygenated haemoglobin (O_2_Hb) and deoxygenated haemoglobin (HHb). Maximum [TSI_max_, (O_2_Hb)_max_, (HHb)_max_] and minimum values [TSI_min_, (O_2_Hb)_min_, (HHb)_min_] were calculated for each occlusion/reperfusion cycle. Area Under the Curve (AUC) was calculated for each occlusion using absolute values [TSI_AUC_, (O_2_Hb)_AUC_, (HHb)_AUC_, ΔHb_AUC_], in order to assess the magnitude of oxygenation variations during deoxygenation and reoxygenation (Agbangla et al., 2017; de Oliveira et al., 2021; Manfredini et al., 2009). The AUC for reoxygenation was calculated from the zero-crossing closest to each of the three hyperemia spikes to the first crossing of 63% deoxygenation after each spike. Repeated occlusions may alter microcirculation, causing oxygenation to remain elevated above baseline (Kraemer et al., 2011). The 63% threshold was chosen to ensure consistent comparisons between participants. The time constant (τ) was calculated for [HHb] as the time taken from the beginning of each occlusion to reach 63% of the maximal [HHb] value. The NIRS-derived parameters from the three cycles were averaged to obtain a single value per parameter for each session, as intra-session muscle oxygenation assessments using CW-NIRS are reliable (Desanlis et al., 2022).

**FIGURE 3:**
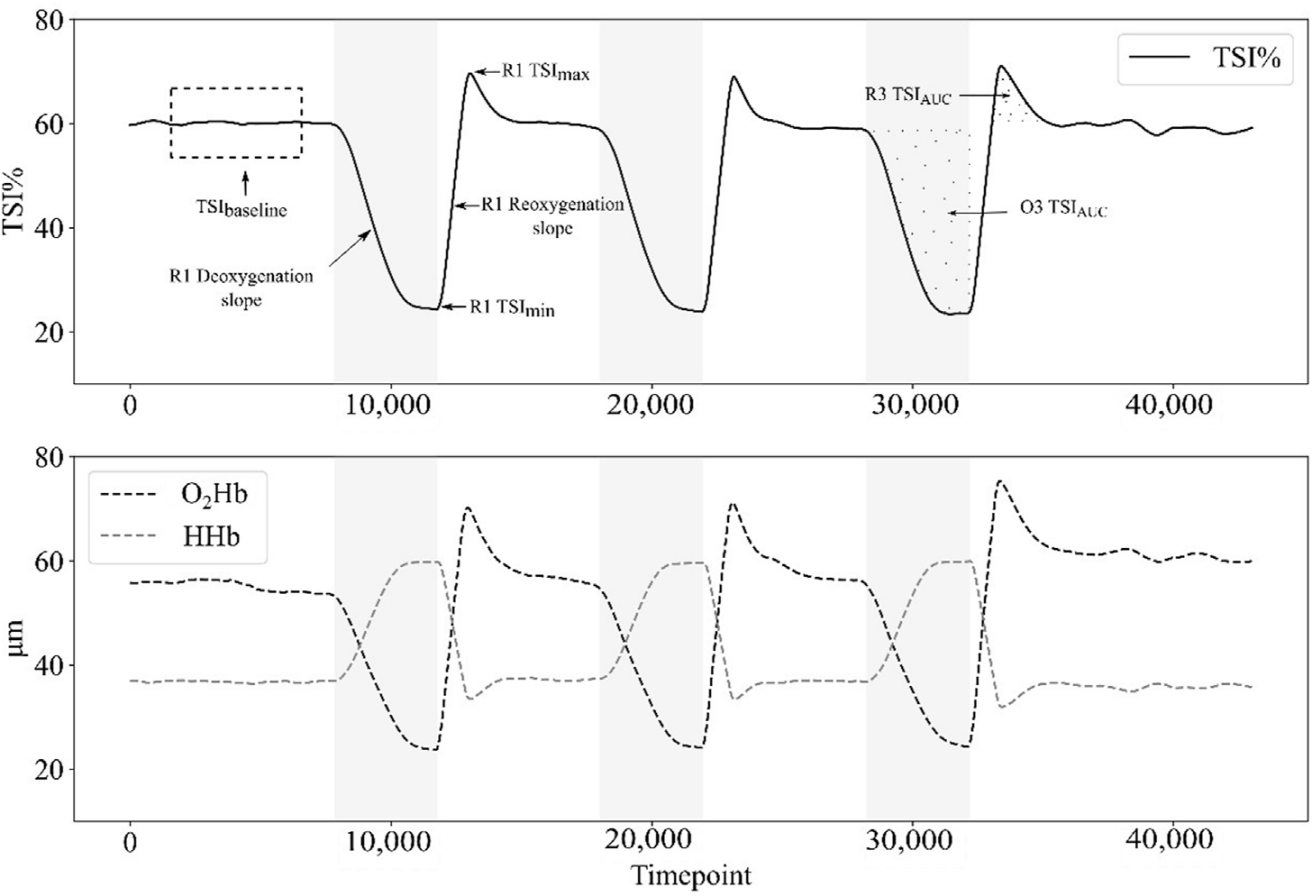
Example of tissue saturation index (TSI%), oxyhemoglobin concentration [(O_2_Hb)] and deoxyhemoglobin concentration [(HHb)] responses during the protocol for a single participant in group 3 (G3: 250 mmHg). The dashed rectangle represents the last minute at baseline, which is averaged to provide the baseline value of each parameter. When the cuff is inflated during occlusion phases (O1, O2, O3; in grey on the graph), both TSI% and [O_2_Hb] decrease until their minimum, whereas [HHb] increases to its maximum. When the cuff is deflated at the beginning of reperfusions phases (R1, R2, R3), TSI% and [O_2_Hb] rise until their maximum above the baseline value (hyperemia spike), whereas [HHb] reach its minimum. During occlusion phases and post-occlusive reactive hyperemia phases, the area under the curve (AUC) is calculated.

Deoxy – and reoxygenation slopes were calculated for each parameter [TSI_slope_, (HHb)_slope_, (O_2_Hb)_slope_ and ΔHb_slope_] as the slope of TSI, [HHb], [O_2_Hb], and ΔHb during the first 30 s of the deoxy – or reoxygenation curve (Figure 3) (Papadopoulos et al., 2018). The start, the end, and the peak value of the deoxy – or reoxygenation curve were calculated using an automatic peaks detection Python routine, with a 10% threshold for both start and end (Python 3.8.8). The data analyzed in this study is part of a larger dataset previously published to assess intra- and inter-session NIRS device reliability (Desanlis et al., 2022). This study provides original findings on muscle oxygenation responses to arterial occlusion.

### 2.5 Statistical analysis

Data are presented as means ± standard deviations (SD). Outliers deviating from mean ± 2 SD were removed from the dataset (Bhambhani et al., 2006; Southern et al., 2014). After testing for normality (Shapiro-Wilk’s test), the homogeneity of variance (Levene’s test) and the sphericity (Mauchly’s test), mixed analysis of variance (mixed ANOVA) with Bonferroni *post hoc* correction were used to determine the difference between groups. One-way ANOVA was used to compare demographical data between groups. The magnitude of effects was calculated with Cohen’s d and interpreted as small (0.2 ≥ d > 0.5), medium (0.5 ≥ d > 0.8), and large (d ≥ 0.8) (Cohen, 1988). Non-parametric analysis of variance (Kruskal-Wallis test) with Dunn’s *post hoc* were used when *p*-value of Shapiro-Wilk’s and Levene’s tests were lower than significance threshold. Effect sizes for non-parametric *post hoc* were given by the rank biserial correlation (r) for the Mann-Witney test (Kerby, 2014), as r = 0 for an absence of relationship between the two groups, and r = 1 for the strongest difference in ranks between groups. Statistical analysis was performed using JASP 0.17.1.0 (JASP Team, 2023). Statistical significance was set at *P* < 0.05.

## 3 Results

### 3.1 Environmental and participant descriptions

Four participants were excluded due to technical issues. Seven participants were excluded during data analysis due to inconsistent NIRS responses (i.e., tissue saturation index reaching 0%; prolonged degraded signal quality). Thus, thirty-five young males were included in the study.

Non-significant differences were observed across all variables of participants’ characteristics (Table 1). No significant humidity differences were found between groups in S1 (*p* = 0.33) or S2 (*p* = 0.54) and between sessions (S1: 41% ± 14%, S2: 45% ± 15%, *p* = 0.095). No significant temperature differences were found between groups in S1 (*p* = 0.24) or S2 (*p* = 0.20) and between sessions (S1: 21.2°C ± 1.8°C, S2: 21.3% ± 1.5%, *p* = 0.96).

### 3.2 Effects of occlusion pressure on muscle deoxygenation

**FIGURE 4:**
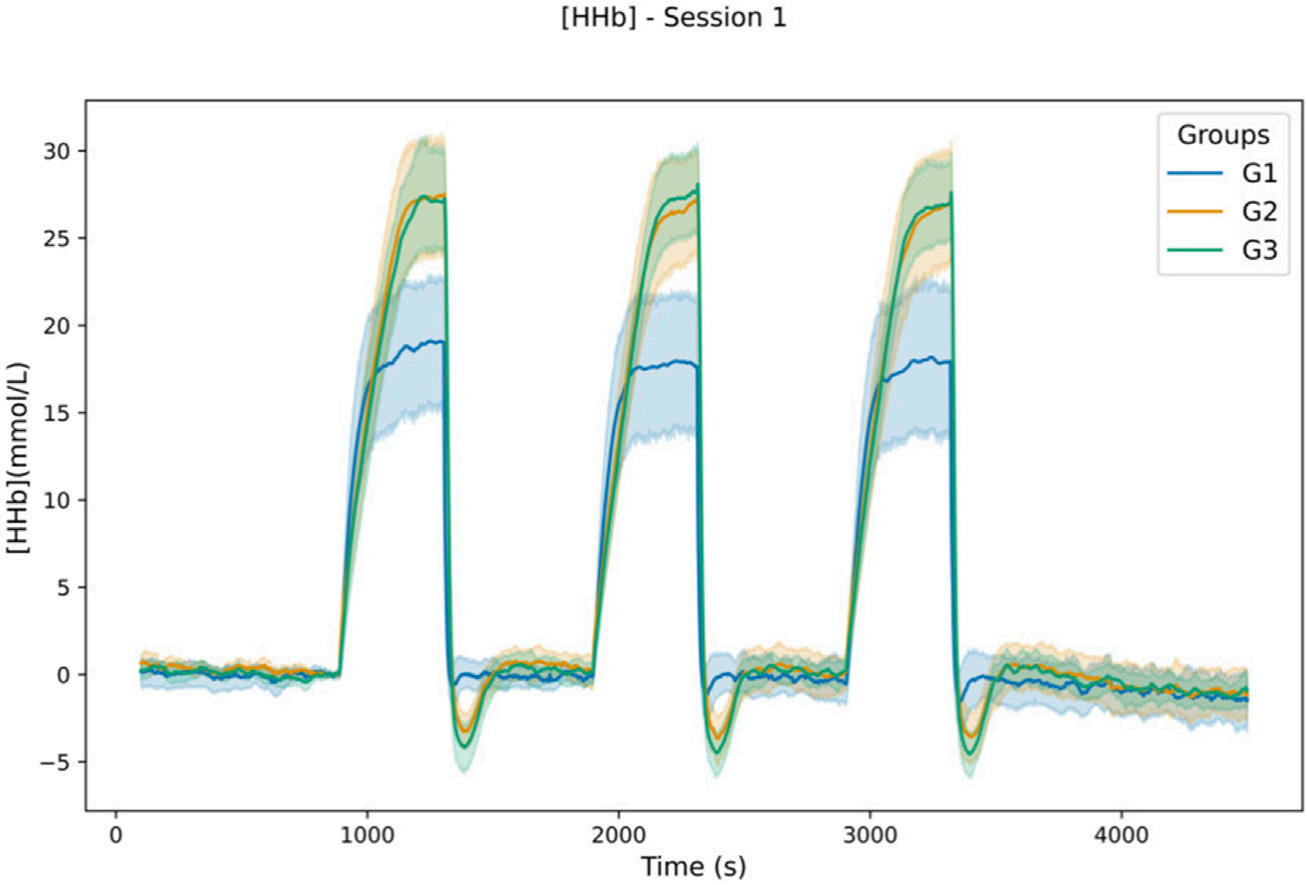
Lineplot of [HHb] response during the protocol in S1. Values are aggregate over participants of each group to plot the mean (in bold) and 95% confidence interval using seaborn library for python (Waskom, 2021).

NIRS-derived parameters showed that using absolute (G3) or individualized (G2) occlusion pressures over SBP induced significantly greater deoxygenation compared to venous occlusion (G1), in terms of maximum values reached during occlusion, as reported in Table 2. A significant difference was shown for TSI_min_ in both sessions between G1 and G2, G1 and G3, and between G2 and G3 only for session 2. Regarding [O_2_Hb], a significant difference was found using Dunn’s *post hoc* comparisons between G1 and G2 (*p* < .001), G1 and G3 (*p* < .001), but not between G2 and G3 in both sessions. [HHb]_max_ during occlusions showed a significant difference for both sessions between G1 and G2 (*p* < .001), between G1 and G3 (*p* < .001), but not between G2 and G3 (Figure 4).

**TABLE 2:** Changes in NIRS-derived parameters during occlusion.

Variable	Session	A	B	A: mean ± SD	B: mean ± SD	P_bonf_	Effect size
TSI_min_	S1	G1	G2	−7.688 ± 5.500	−31.797 ± 5.914	<0.001*	d = 4.067
(%)	S1	G1	G3	−7.688 ± 5.500	−34.482 ± 5.487	<0.001*	d = 4.520
	S1	G2	G3	−31.797 ± 5.914	−34.482 ± 5.487	0.735	d = 0.453
	S2	G1	G2	−11.782 ± 3.295	−28.094 ± 5.271	<0.001*	d = 3.109
	S2	G1	G3	−11.782 ± 3.295	−34.589 ± 6.367	<0.001*	d = 4.347
	S2	G2	G3	−28.094 ± 5.271	−34.589 ± 6.367	0.015*	d = 1.238
[O2Hb]_min_	S1	G1	G2	16.571 ± 13.588	−19.308 ± 11.005	<0.001*	r = 1.000
(mmol/L)	S1	G1	G3	16.571 ± 13.588	−17.828 ± 6.941	<0.001*	r = 1.000
	S1	G2	G3	−19.308 ± 11.005	−17.828 ± 6.941	1.000	r = 0.024
	S2	G1	G2	12.972 ± 10.05	−17.318 ± 7.561	<0.001*	r = 1.000
	S2	G1	G3	12.972 ± 10.05	−19.708 ± 4.982	<0.001*	r = 1.000
	S2	G2	G3	−17.318 ± 7.561	−19.708 ± 4.982	1.000	r = −0.051
[HHb]_max_	S1	G1	G2	18.924 ± 6.367	29.145 ± 4.823	<0.001*	d = −1.899
(mmol/L)	S1	G1	G3	18.924 ± 6.367	28.509 ± 4.893	<0.001*	d = −1.780
	S1	G2	G3	29.145 ± 4.823	28.509 ± 4.893	1.000	d = 0.118
	S2	G1	G2	17.837 ± 4.428	26.196 ± 3.218	<0.001*	d = −2.025
	S2	G1	G3	17.837 ± 4.428	29.186 ± 4.43	<0.001*	d = −2.749
	S2	G2	G3	26.196 ± 3.218	29.186 ± 4.43	0.199	d = −0.724

Values are reported as mean ± SD; TSI, tissue saturation index (%); O_2_Hb and HHb, oxy – and deoxyhemoglobin; A and B, names of the first and the second group of the condition in the pairwise t-test; *, significant difference between groups; Effect sizes, Cohen’s d and rank biserial correlation (r).

This greater deoxygenation during occlusion for participants of G2 and G3 is also confirmed by the higher area under the curve (AUC) values, in TSI_AUC_ and ΔHb_AUC_, for G2 and G3 compared to G1, without significant differences between G2 and G3, as reported in Table 3.

**TABLE 3:** Quantity of deoxygenation during occlusion.

Variable	Session	A	B	A: mean ± SD	B: mean ± SD	P_bonf_	Effect size	
TSI_AUC_	S1	G1	G2	−1959.235 ± 1417.399	−10908.097 ± 1607.469	<0.001*	d = 5.231	
(a.u.)	S1	G1	G3	−1959.235 ± 1417.399	−11079.344 ± 1828.094	<0.001*	d = 5.331	
	S1	G2	G3	−10908.097 ± 1607.469	−11079.344 ± 1828.094	1.000	d = 0.100	
	S2	G1	G2	−3563.863 ± 1040.148	−10066.303 ± 2367.666	<0.001*	d = 3.173	
	S2	G1	G3	−3563.863 ± 1040.148	−10784.103 ± 2156.969	<0.001*	d = 3.523	
	S2	G2	G3	−10066.303 ± 2367.666	−10784.103 ± 2156.969	1.000	d = 0.350	
ΔHb_AUC_	S1	G1	G2	1130.178 ± 2501.658	−15513.846 ± 5187.309	<0.001*	d = 4.437	
(a.u.)	S1	G1	G3	1130.178 ± 2501.658	−13839.073 ± 2034.38	<0.001*	d = 3.99	
	S1	G2	G3	−15513.846 ± 5187.309	−13839.073 ± 2034.38	0.854	d = −0.446	
	S2	G1	G2	−1540.371 ± 2053.732	−14855.142 ± 4222.848	<0.001*	d = 3.967	
	S2	G1	G3	−1540.371 ± 2053.732	−14757.584 ± 2655.487	<0.001*	d = 3.938	
	S2	G2	G3	−14855.142 ± 4222.848	−14757.584 ± 2655.487	1.000	d = −0.029	

Values are reported as mean ± SD; TSI, tissue saturation index (%); Δ[Hb], haemoglobin difference; AUC, area under the curve; a.u., arbitrary unit; A and B, names of the first and the second group of the condition in the pairwise t-test; *, significant difference between groups; Effect sizes, Cohen’s d and rank biserial correlation (r).

The deoxygenation, while greater for G2 and G3, was also faster, as indicated by the large difference in [HHb] time constant (τ) [(HHb)τ], between G2 and G1, and between G3 and G1 (Table 4). The TSI τ during occlusion showed significant differences with large effect sizes in both sessions between G1 and G2, G1 and G3, but not between G2 and G3 (Table 4).

**TABLE 4:** Speed and length of deoxygenation after occlusion.

Variable	Session	A	B	A: mean ± SD	B: mean ± SD		P_bonf_	Effect size
TSI τ	S1	G1	G2	77.275 ± 39.991	128.728 ± 19.664		<0.001*	d = −1.924
(s)	S1	G1	G3	77.275 ± 39.991	148.247 ± 21.93		<0.001*	d = −2.654
	S1	G2	G3	128.728 ± 19.664	148.247 ± 21.93		0.253	d = −0.730
	S2	G1	G2	89.763 ± 15.633	116.794 ± 23.457		0.040*	d = −1.202
	S2	G1	G3	89.763 ± 15.633	131.397 ± 25.059		<0.001*	d = −1.851
	S2	G2	G3	116.794 ± 23.457	131.397 ± 25.059		0.368	d = −0.649
TSI_slope_	S1	G1	G2	−0.044 ± 0.069	−0.145 ± 0.027		0.006*	r = 0.852
(%/s)	S1	G1	G3	−0.044 ± 0.069	−0.153 ± 0.034		0.002*	r = 0.829
	S1	G2	G3	−0.145 ± 0.027	−0.153 ± 0.034		1.000	r = 0.103
	S2	G1	G2	−0.087 ± 0.048	−0.141 ± 0.034		0.010*	d = 1.332
	S2	G1	G3	−0.087 ± 0.048	−0.159 ± 0.031		<0.001*	d = 1.759
	S2	G2	G3	−0.141 ± 0.034	−0.159 ± 0.031		0.889	d = 0.427
[HHb] τ	S1	G1	G2	57.759 ± 14.909	140.633 ± 16.781		<0.001*	d = −3.791
(s)	S1	G1	G3	57.759 ± 14.909	146.542 ± 26.458		<0.001*	d = −4.062
	S1	G2	G3	140.633 ± 16.781	146.542 ± 26.458		1.000	d = −0.270
	S2	G1	G2	68.733 ± 6.088	140.767 ± 16.436		0.002*	r = −1.000
	S2	G1	G3	68.733 ± 6.088	148.989 ± 22.158		<0.001*	r = −1.000
	S2	G2	G3	140.767 ± 16.436	148.989 ± 22.158		1.000	r = −0.212

Values are reported as mean ± SD; TSI, tissue saturation index (%); HHb, deoxyhemoglobin; τ, time constant; A and B, names of the first and the second group of the condition in the pairwise t-test; *, significant difference between groups; Effect sizes, Cohen’s d and rank biserial correlation (r).

As reported in Table 4, the faster deoxygenation for G2 and G3 was confirmed by steeper TSI_slopes_ between G1 and G2, and between G1 and G3, without significant differences between G2 and G3.

### 3.3 Effects of occlusion pressure on muscle reoxygenation

**FIGURE 5:**
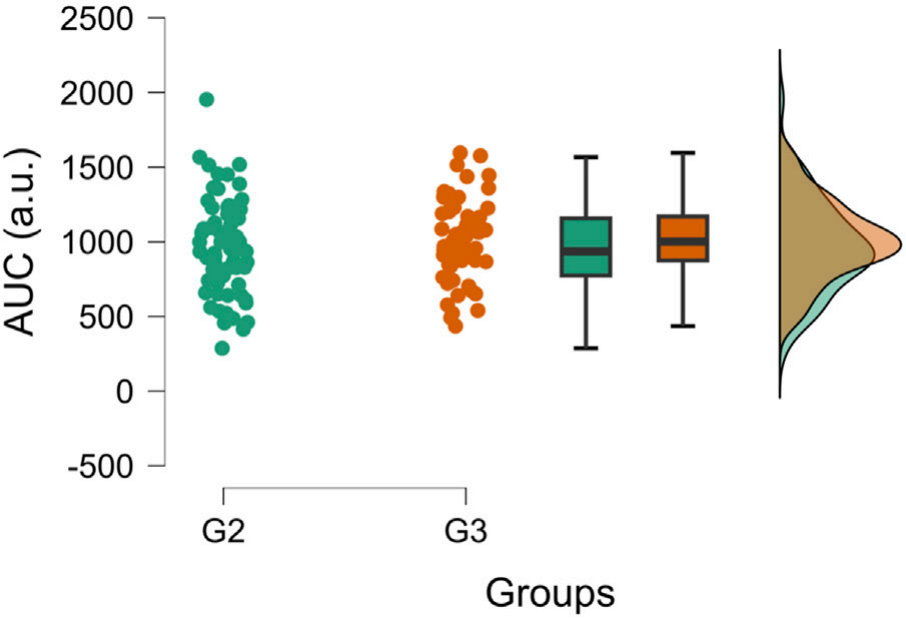
Raincloud plots of TSI_AUC_ during PORH. The plot highlights the lack of differences between group 2 (G2, n = 12) and group 3 (G3, n = 12) during PORH. The values from S1 and S2 are displayed. Generated using JASP 0.17.1.0 (JASP Team, 2023).

After cuff deflation, muscle oxygenation of the occluded arm increased rapidly and overcame the baseline level for a short duration. This post-occlusive reactive hyperemia (PORH) is affected by the pressure applied during occlusion since a significant effect of group on ΔTSI_max_ reached during hyperemia spike was found for both sessions between G1 and G2, G1 and G3, but not between G2 and G3, with higher PORH values for higher pressures (Table 5). In addition, no significant differences were found between G2 and G3 during PORH for TSI_AUC_ (G2 = 961.425 ± 296.556 a.u.; G3 = 1007.45 ± 228.827 a.u.; *p* = 0.559) (Figure 5) and TSI τ (G2 = 15.267 ± 6.064s; G3 = 14.779 ± 4.899 s; *p* = 0.697).

**TABLE 5:** Changes in TSI% after cuff deflation.

Variable	Session	A	B	A: mean ± SD	B: mean ± SD	P_bonf_	Effect size
TSI_max_	S1	G1	G2	1.933 ± 2.01	9.54 ± 3.274	0.006*	r = −0.981
(%)	S1	G1	G3	1.933 ± 2.01	12.219 ± 1.485	<0.001*	r = −1.000
	S1	G2	G3	9.54 ± 3.274	12.219 ± 1.485	0.349	r = −0.497
	S2	G1	G2	1.831 ± 1.627	10.179 ± 2.33	<0.001*	d = −4.739
	S2	G1	G3	1.831 ± 1.627	10.734 ± 1.046	<0.001*	d = −5.055
	S2	G2	G3	10.179 ± 2.33	10.734 ± 1.046	1.000	d = −0.315

Values are reported as mean ± SD; TSI, tissue saturation index (%); AUC, area under the curve; A and B, names of the first and the second group of the condition in the pairwise t-test; *, significant difference between groups; Effect sizes, Cohen’s d and rank biserial correlation (r).

Regarding the speed of reperfusion during PORH, G2 and G3 pressures tend to induce faster reperfusion than G1. [HHb]_slope_ during reperfusion was significantly steeper for G2 compared to G1, and for G3 compared to G1, without significant differences between G2 and G3 (Table 6). However, a tendency of a steeper slope for G3 compared to G2 could be highlighted in session 2. When TSI_slopes_ of both sessions are averaged for G2 and G3 and compared with a t-test, a significant difference between groups is shown (1.264 ± 0.147 vs. 1.044 ± 0.209, *p =* 0.010, d = −1.184).

**TABLE 6:** Reperfusion speed after cuff deflation.

Variable	Session	A	B	A: mean ± SD	B: mean ± SD	P_bonf_	Effect size
TSI_slope_	S1	G1	G2	0.265 ± 0.103	1.119 ± 0.228	0.003*	r = −1.000
(%/s)	S1	G1	G3	0.265 ± 0.103	1.282 ± 0.182	<0.001*	r = −1.000
	S1	G2	G3	1.119 ± 0.228	1.282 ± 0.182	0.598	r = −0.397
	S2	G1	G2	0.336 ± 0.122	0.936 ± 0.265	0.008*	r = −0.968
	S2	G1	G3	0.336 ± 0.122	1.258 ± 0.130	<0.001*	r = −1.000
	S2	G2	G3	0.936 ± 0.265	1.258 ± 0.130	0.079	r = −0.701
[HHb]_slope_	S1	G1	G2	−0.392 ± 0.137	−0.931 ± 0.124	<0.001*	d = 3.628
(%/s)	S1	G1	G3	−0.392 ± 0.137	−0.946 ± 0.154	<0.001*	d = 3.730
	S1	G2	G3	−0.931 ± 0.124	−0.946 ± 0.154	1.000	d = 0.102
	S2	G1	G2	−0.398 ± 0.12	−0.854 ± 0.168	<0.001*	d = 3.154
	S2	G1	G3	−0.398 ± 0.12	−0.937 ± 0.131	<0.001*	d = 3.729
	S2	G2	G3	−0.854 ± 0.168	−0.937 ± 0.131	0.485	d = 0.574

Values are reported as mean ± SD; TSI, tissue saturation index (%); HHb, deoxyhemoglobin; A and B, names of the first and the second group of the condition in the pairwise t-test; *, significant difference between groups; Effect sizes, Cohen’s d and rank biserial correlation (r).

## 4 Discussion

The aim of this study was to evaluate the peripheral hemodynamic responses to partial and complete, absolute, or individualized blood-flow occlusions on the arm in the rested state. The results support our hypothesis that the magnitude of hemodynamic changes during occlusion and reperfusion are greater for groups with complete arterial occlusion, without differences between absolute or individualized pressure group.

### 4.1 Effects of occlusion pressure on deoxygenation

Data showed greater deoxygenation induced by suprasystolic pressures compared to G1 but cannot show differences between both groups with occlusion pressures over the SBP (G2 and G3). This greater deoxygenation reached for G2 and G3 is characterized by higher values of [HHb]_AUC_, [HHb]_max_ (Figure 3) and lower values of TSI_min_ compared to G1, with no differences between G2 and G3, except for TSI_min_ in S2. In addition, the deoxygenation rate is not different between G2 and G3, while it is significantly faster for both groups than for G1.

These present results support the hypothesis that the hemodynamic response is driven by occlusion pressure when comparing partial and complete occlusions. However, no systematic differences were found between G2 and G3, indicating that no dose-response effect could be elicited for pressures over the SBP. The hemodynamic response seems similar between suprasystolic pressures. Thus, similar effects on deoxygenation could be induced at a much lower intensity than IPC pressures established in the literature (i.e., between 200 and 250 mmHg) (O’Brien and Jacobs, 2021).

Our findings are attractive since a dose-response effect was previously found in both upper and lower limbs with occlusion pressures of 140, 160 and 180 mmHg (Cunniffe et al., 2017), with greater deoxygenation characterized by a greater drop in [O_2_Hb] after occlusion with higher pressures. However, the authors reported that 140 and 160 mmHg pressures were insufficient to induce complete arterial occlusion in the upper limb for all participants (Cunniffe et al., 2017; Sharma et al., 2014). Thus, the results reported in the study from Cunniffe et al. (2017) support our findings since the authors found greater deoxygenation for complete (180 mmHg) and partial arterial occlusion (140 and 160 mmHg).

Since arm arterial occlusion depends on SBP, which is highly variable between individuals, and arm circumference in a lesser extent (Loenneke et al., 2015), using absolute values for IPC cannot guarantee complete arterial occlusion (Pickering et al., 2005; Sharma et al., 2014). Thus, it was proposed to use occlusion pressures based on the SBP. Some studies used individualized occlusion pressures ranging from 10 to 50 mmHg over the SBP (O’Brien and Jacobs, 2021). However, SBP measurements could show significant shortcomings, leading to the misclassification of patients (Pickering et al., 2005). Moreover, the first Korotkoff sound, attributed to systolic pressure, tends to underestimate the systolic pressure recorded by direct intra-arterial measurement up to 31 mmHg (Hunyor et al., 1978; Pickering et al., 2005). Thus, choosing an individualized pressure of less than 50 mmHg > SBP cannot guarantee complete arterial occlusion as well.

Even with an absence of a detectable pressure pulse, Mouser et al. (2017) reported a minor blood flow in several participants, which could be attributed to enhanced BP caused by solicitation of muscle metaboreflex and mechanoreflex or blood reaching occluded tissues through deep arteries (Brown et al., 2018; Spranger et al., 2015). Moreover, subtle movements of the participant could reduce pressure underneath the cuff leading to incomplete occlusion (Brown et al., 2018). Light postural changes could be caused by discomfort induced by cuff pressure. Previous studies have shown that IPC induces a discomfort in both upper and lower limbs (ratings of pain on a 0–10 scale during IPC: 4 ± 0.7 and 4.36 ± 1.29 for the arm (Lalonde and Curnier, 2015; Sharma et al., 2014); 7 ± 0.4 for the legs (Sharma et al., 2014)). Thus, it is possible that the discomfort caused by the arterial occlusion led to postural changes during the protocol, although the participants were asked to stay immobile.

### 4.2 Effects of occlusion pressure on reoxygenation speed

Cuff deflation induces a fast reoxygenation – or reperfusion – phase caused by restored blood inflow. Our data showed that this phase was faster for G2 and G3 than for G1, with steeper reperfusion slopes. No significant differences in TSI_slope_ for reoxygenation rate were found between G2 and G3 when sessions were compared independently but a significant difference was found when measurements of the two sessions were averaged for G2 and G3. A greater pressure used to occlude blood flow may induce a faster reoxygenation response in the 30 s window following cuff deflation, which could be due to an increased blood flow after deflation.

It has been shown that IPC decreases blood pH and increases blood lactate concentration ([La^−^]), during the occlusion, at much lower occlusion pressures than pressures used in the current study (Sharma et al., 2014). An enhanced clearance, or a lower production of [La^−^], has been shown during exercise preceded by IPC (Bailey et al., 2012b), which could result from better endothelial protection induced by IPC (Gu et al., 2021; Rajendran et al., 2013). Better endothelial protection enhances vascular function, which regulates the blood flow to remove and transport lactate. Increased blood flow could also arise from increased adenosine levels and the opening of ATP-sensitive potassium (K_ATP_) channels under hypoxia conditions (Bailey et al., 2012a; Riksen et al., 2004; Rosenberry and Nelson, 2020), that belongs to the main mediators of PORH (Mahé et al., 2012). Indeed, inwardly rectifying potassium channels and Na+/K+-ATPase are the main determinants of the magnitude of PORH in humans (Crecelius et al., 2013). After multiple ischemia-reperfusion cycles, it has been shown that capillary blood flow was significantly increased (Kraemer et al., 2011).

Interestingly, the difference in reperfusion speed between G2 and G3 could also result from greater vasodilation inducing a higher blood flow (Enko et al., 2011). In addition, the velocity of red blood cells (calculated by assessing the distance of red blood cells movement in each frame) is increased after the IPC procedure in an animal model (Tapuria et al., 2009), which could explain the result found in the present study. However, this higher red blood cells velocity has not been demonstrated in muscle (Adanali et al., 2002).

Even though the TSI_slope_ of G3 was steeper than the slope of G2, it is noteworthy that this difference in our result is only shown for TSI but not for [HHb] or [O_2_Hb]. Since TSI is calculated from [O_2_Hb], which is known to be sensitive to blood flow changes, this parameter may not be the most useful to characterize reoxygenation. Thus, it would be fallacious to conclude that reoxygenation is faster when higher pressures are used after a complete arterial occlusion. These findings need to be investigated in future studies to confirm or not our results.

### 4.3 Effects of occlusion pressure on PORH

Arterial occlusion induces a transient phase of fast reperfusion known as post-occlusive reactive hyperemia (PORH) when the cuff is deflated after a sufficient time. The PORH represents the magnitude of limb reperfusion after ischemia (Rosenberry and Nelson, 2020), and PORH assessment using NIRS has been largely reported in the literature (Rosenberry and Nelson, 2020). Our study compared the TSI_AUC_ of PORH between G2 and G3. No differences were found between both groups. Similarly, while G2 showed longer PORH phases in both sessions than G3, this difference was not significant. Thus, although reoxygenation seems to be faster for G3 than G2, the magnitude of the reperfusion, characterized by AUC, is not significantly different between groups (Figure 5). Therefore, this conclusion supports the necessity of being cautious when concluding about the speed of reperfusion of TSI between G3 and G2. It is then necessary to not only look at the speed of reperfusion but also take into account the other parameters such as the AUC, and maximums or minimums reached during occlusion and reperfusion phases.

These findings are consistent with previous results that could not show differences in PORH values between 140, 160 and 180 mmHg, sweeping any dose-response effect away (Cunniffe et al., 2017). Occlusion in this study led to PORH even with much lower pressures than traditionally used. It is also consistent with our results that cannot show significant differences between the two groups with complete arterial occlusion.

## 5 Limitations

A limitation of this study is that room temperature was not controlled, which could affect skin temperature (Hom et al., 2004). However, no significant differences between groups or sessions have been found. In addition, as stated previously, few studies using occlusion pressures over the SBP reported incomplete arterial occlusion. The gold standard to assess the absence of pulse is the Doppler ultrasound (Brown et al., 2018), which was not used in our study. However, the pressures selected in the current study are consistent with the literature (O’Brien and Jacobs, 2021) and high enough to overcome SBP measurement errors (Pickering et al., 2005) in young healthy participants, or decreases in cuff pressure due to subtle postural changes (Brown et al., 2018).

Another limitation of this study is the lack of ATT assessment at the NIRS site. Including participants with a fat layer greater than one-quarter the distance between the NIRS receptor and the most distant emitter could have affected the signal (Barstow, 2019). This may explain the inconsistent NIRS responses observed in seven participants.

As mentioned in the introduction, typical ischemic preconditioning (IPC) interventions involve periods of ischemia lasting between 5 and 10 min (O’Brien and Jacobs, 2021). In this study, the duration of ischemia periods was set at 7 min, which was also found in only one other study conducted on humans (Bushell et al., 2002). Initially, this specific duration was chosen to investigate the effects of occlusion pressure on heart rate variability (HRV) and blood pressure variability (BPV), requiring a minimum of 256 points for analysis (Souza Neto et al., 2003). However, due to a technical issue during data collection, HRV and BPV cannot be presented in this article.

## 6 Conclusion

This study presents compelling evidence that individualized occlusion pressures (SBP + 50 mmHg), within the healthy population, yield hemodynamic responses measured by NIRS, that are not significantly different from those seen with traditionally higher absolute pressures (>200 mmHg) reported in the literature. With the exception of the reoxygenation rate, no significant differences were observed between G2 and G3. Using more personalized IPC protocols can benefit athletes and patients by improving performance, blood flow, and oxygen delivery while offering a safer approach than using high absolute pressures (Marocolo et al., 2016; Marocolo et al., 2019; Paradis-Deschênes et al., 2016). The use of NIRS devices to monitor muscle oxygenation allows for precise, real-time, and non-invasive interventions, applicable in research, clinical, and training contexts (Perrey, 2022; Perrey and Ferrari, 2018). Overall, these insights support the standardization of IPC protocols, making them more consistent and reliable across various applications (O’Brien and Jacobs, 2021). These findings highlight the practicality and effectiveness of using individualized occlusion pressures based on systolic blood pressure (SBP) rather than relying on excessively high absolute pressures. By challenging the conventional practice, this approach offers an alternative for optimizing occlusion pressure protocols while ensuring participant safety in research and clinical settings.

## Data Availability

The datasets presented in this study can be found in online repositories. The names of the repository/repositories and accession number(s) can be found below: https://doi.org/10.5281/zenodo.7920956.
